# Prehospital computed tomography in a rural district for rapid diagnosis and treatment of stroke

**DOI:** 10.1177/23969873241267084

**Published:** 2024-09-28

**Authors:** Jørgen Ibsen, Maren Ranhoff Hov, Gunn Eli Tokerud, Julia Fuglum, Marianne Linnerud Krogstad, Marie Stugaard, Hege Ihle-Hansen, Christian Georg Lund, Christian Hall

**Affiliations:** 1 Department of Medicine, Ringerike Hospital, Vestre Viken Hospital Trust, Honefoss, Norway; 2 The Norwegian Air Ambulance Foundation, Oslo, Norway; 3 Oslo Metropolitan University, Bachelor of Paramedic, Oslo, Norway; 4 Department of Medical Diagnostics, Ringerike Hospital, Vestre Viken Hospital Trust, Honefoss, Norway; 5 Department of Neurology, Lillehammer Hospital, Sykehuset Innlandet Hospital Trust, Lillehammer, Norway; 6 Vikersund Bad Rehabilitation Centre, Helse Sør-Øst, Vikersund, Norway; 7 Institute for Surgical Research, University of Oslo, Oslo, Norway; 8 Department of Neurology, Oslo University Hospital, Oslo, Norway; 9 Institute of Clinical Medicine, University of Oslo, Oslo, Norway

**Keywords:** prehospital, computed tomography, stroke, thrombolysis, telestroke

## Abstract

**Background:**

Early diagnosis and triage of patients with ischemic stroke is essential for rapid reperfusion therapy. The prehospital delay may be substantial and patients from rural districts often arrive at their local hospital too late for disability-preventing thrombolytic therapy due to prolonged transport times.

**Methods:**

Hallingdal District Medical Centre (HDMC) is located in a rural area of Norway and is equipped with a computed tomography (CT) scanner. We established emergency pathways of CT imaging and thrombolytic treatment of patients with acute ischemic stroke at HDMC. During office hours these pathways were managed by a radiographer and a general physician supported by videoconference from the Primary Stroke Centre. Outside office hours we remotely controlled the CT exam and supported telestroke guided paramedics handling and examining the patients. With a primary aim of demonstrating the feasibility of this de novo concept we enrolled patients in the period 2017–2021 into a comparative cohort observational study. We compared patients treated at HDMC (the Rural CT group) to patients from two other rural regions in Norway with similar distances to their local hospital but without access to a rural CT scanner (the Reference group).

**Results:**

A total of 86 patients were included in the Rural CT group (mean age 74, 52% male, 43% stroke mimics), and 69 patients were included in the Reference group (mean age 70, 42% male, 28% stroke mimics). Median time from onset of symptoms to completed CT examination was 93 min in the Rural CT group as compared to 240 min in the Reference group (*p* < 0.05). In patients receiving intravenous thrombolysis time from onset of symptoms to treatment was median 124 min in the Rural CT group and 213 min in the Reference group, *p* < 0.05. The frequency of thrombolysis for ischemic stroke did not significantly differ between the two groups.

**Conclusion:**

Combining prehospital rural CT examination with telestroke guided diagnosis and thrombolytic treatment by paramedics may facilitate earlier initiation of thrombolysis for patients with ischemic stroke.

## Graphical abstract

**Graphical abstract:**
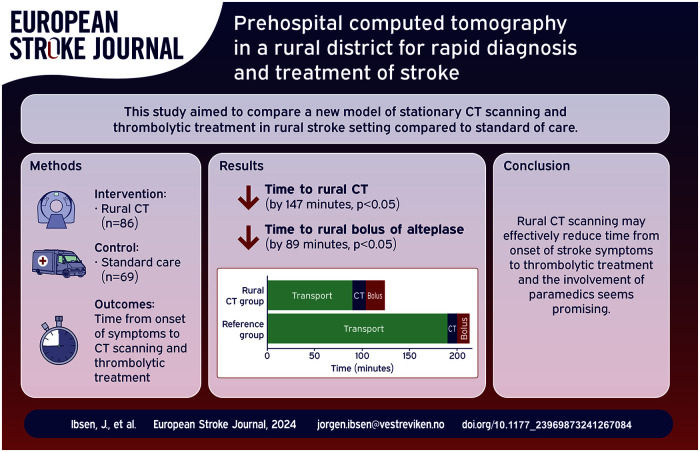

## Introduction

Early reperfusion therapy significantly improves prognosis in patients with acute ischemic stroke.^1–3^ The effectiveness of thrombolysis is time dependent and diminishes rapidly within the first few hours after onset of symptoms. Every minute saved from onset of stroke symptoms to thrombolysis theoretically saves at least 1 day of extra healthy life.^4,5^ Patients with Large Vessel Occlusion (LVO) stroke may benefit substantially from endovascular thrombectomy.^3,6,7^ Prehospital assessment of acute stroke patients has until recently been limited to clinical evaluation of symptom presentation, and the need of early radiological diagnostics has required in-hospital assessment for revascularization.

In 2020, 55% of all Norwegian stroke patients were admitted to the hospital later than 4 h from the onset of stroke.^8^ Consequently, the majority of these patients arrived too late to receive thrombolytic treatment within the time limit of 4.5 h. In Norway, most stroke patients are identified by the prehospital services by first contact to the dispatch center (113) and thereafter assessed clinically by the ambulance service staffed with a 2-person crew, in this paper referred to as paramedics for simplicity.^9^ The Norwegian EMS system has a Helicopter Emergency Medical Service (HEMS) staffed with a pilot, a specialized paramedic and an anesthesiologist.

The development of Mobile Stroke Units (MSU) has introduced cerebral CT scanners in the prehospital environment and thrombolytic therapy may be initiated earlier.^10–12^ Recent European guidelines have recommended MSU for assessment of acute stroke patients,^13^ however the evidence available from rural areas is limited.^14,15^ An alternative solution to the rural transportation disadvantage in acute stroke care is rural deployment of CT equipment operated by paramedics. Traditionally, paramedics do not perform National Institute of Health Stroke Scale (NIHSS) scoring nor administer IV thrombolysis to patients with ischemic stroke. However, a recent Norwegian study demonstrated that paramedics can reliably perform a prehospital NIHSS scoring with the aid of a mobile application and thereby improve the communication with neurologists in Stroke Centers.^16,17^

In this paper, we report results from a proof of concept study in which we hypothesized that rurally stationary CT examination and thrombolytic treatment of ischemic stroke involving telestroke guidance of paramedics is feasible. Our main objective was to investigate a possible reduction in time from onset of symptoms to treatment decision. We report observed effects and compare these to a Reference group from similar rural areas treated by standard practice at their Primary Stroke Centres (PSC).

## Materials and methods

Our study was a comparative cohort observational study comparing two groups of patients. In the Rural CT group patients were enrolled at Hallingdal District Medical Centre (HDMC). In the Reference group patients were enrolled at the local community hospitals of Lillehammer and Kongsberg. HDMC has a 10 beds medical ward, a clinical chemical laboratory and a dialysis unit. The center serves five municipalities with a total population of approximately 25,000 inhabitants. Additionally, the region is a popular tourist destination, attracting around 4.8 million overnight tourists annually.^18^ An ambulance central as well as a HEMS base is located in close proximity to HDMC. In 2016, a CT scanner (General Electric Optima, 660 M40, 64-slice) was installed at the center. HDMC is under the administration of Ringerike Hospital (RH) which is a local community hospital located in Honefoss. The distance between HDMC and RH is 141 km, representing an estimated emergency ambulance transport of 98 min. RH is designated as a PSC. The closest Comprehensive Stroke Centre (CSC) is Oslo University Hospital, located 55 km from RH, an additional 46 min of emergency transport.

During the period from August 1, 2017, to December 31, 2021 patients from the HDMC service area with a prehospitally suspected stroke were enrolled into the study. During working hours (Monday through Friday, 08.00 am–03.30 pm) CT examination was performed by an on-site radiographer. In cases of suspected stroke, patients were directly received in the CT laboratory and the general practitioner at HDMC was via telestroke from RH supervised for neurological examination by the NIHSS and thrombolytic treatment decision with alteplase. The CT scanning included CT Angiography (CTA), and images were interpreted by an on-call radiologist at RH. In case of LVO stroke, eligible patients for thrombectomy were transported directly to the CSC, if possible by HEMS. In both instances thrombolysis was initiated before transport as indicated. Patients suspected for stroke but not eligible for thrombectomy were transported to RH with regular non-urgent transport. In cases of intracerebral hemorrhage, intravenous labetalol was administered to patients with elevated blood pressure (SBP > 140).

**Figure 1.**
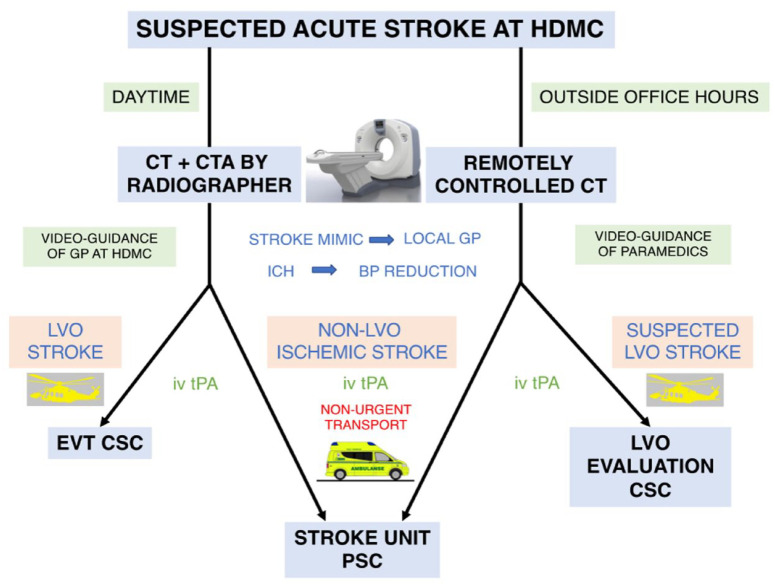
Flowchart of patient diagnostics, treatment and transport logistics of the study period. GP: General Physician; ICH: Intracerebral Hemorrhage; BP: Blood Pressure; iv tPA: Intravenous tissue-type plasminogen activator; LVO: Large Vessel Occlusion; PSC: Primary Stroke Centre; CSC: Comprehensive Stroke Centre; EVT: Endovascular Treatment.

In 2019, the thrombolytic service at HDMC was expanded to a 24 h service including evenings, night shifts and weekends. To compensate for lack of radiographic staff, we recruited 25 locally stationed paramedics to assist during CT examination. They were trained to position patients in the CT scanner, perform neurological assessment using NIHSS and to administer thrombolytic treatment, all under telestroke guidance from RH. During these procedures, the CT scanner was remotely operated by a radiographer at RH. CTA was not available under remote control due to inexact timing of contrast bolus injection. Patients were individually accepted for direct transport to CSC for LVO evaluation based on pre-stroke morbidity (mRS < 2), duration and severity of symptoms (NIHSS > 5), as well as the non-contrast CT images. Figure 1 illustrates patient diagnostics, treatment and transport logistics at HDMC.

**Figure 2.**
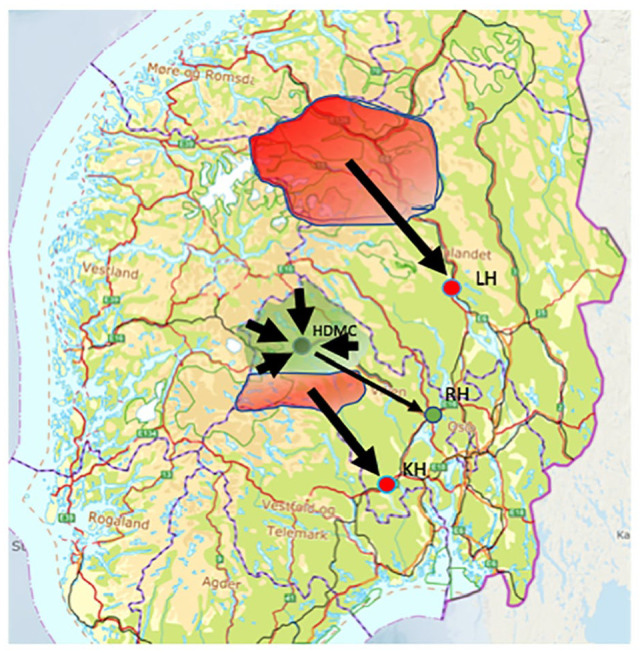
Southern Norway. The green area (Hallingdal) represents the catchment area for the Rural CT group and the location of HDMC and RH (green dots). The red areas (Numedal, Gudbrandsdal) represent the catchment areas for reference (Reference group). PSCs in these areas, Lillehammer Hospital (LH) and Kongsberg Hospital (KH), are marked by red dots.

Patients in the Reference group were enrolled from geographical areas in the southern part of Norway without access to a rural CT service. These areas included six municipalities in Gudbrandsdal and one municipality in Numedal, in which the population had approximately equal transport distances to their respective primary stroke centre hospitals as the population in Hallingdal, Figure 2. In these municipalities, patients with suspected stroke were as a 24 h routine transported by ambulance to their PSCs at Lillehammer Hospital (LH) and Kongsberg Hospital (KH) for CT examination (including CTA) and assessment. The distance from the area with the highest population density in Gudbrandsdal (Otta) to LH is 109 km (67 min of emergency ambulance transport). The distance from Nore in Numedal to KH is 81 km (58 min of emergency transport). Oslo University Hospital is the CSC also for these hospitals, constituting an additional 186 km (99 min) and 81 km (50 min) of transport respectively.

### Patient recruitment

Acute stroke was suspected by paramedics based on at least one persistent symptom according to the FAST mnemonic, frequently after consulting the on-call physician at the PSC. All patients over 18 years undergoing CT examination within the first 24 h after onset of stroke symptoms were included prospectively after informed consent. Data were collected from the electronic medical records during the hospital stay. In August 2018, secondary to limited recruitment into the Reference group, we amended the protocol to allow for retrospective collection of data. Patients were identified from electronic medical records for stays during the study period and asked by letter for informed consent. 15% of the patients in the Rural CT group and 42% in the Reference group were thus registered retrospectively. Patients that had a discharge diagnosis of stroke were followed up by telephone after 3 months for modified Rankin Scale (mRS) scoring. In the retrospectively included patients 3 months mRS score was registered when available in hospital records.

Patients with “wake-up stroke” or unknown onset of symptoms were excluded. Similarly, patients showing rapid and total regress of symptoms (less than 30 min) consistent with Transient Ischemic Attack (TIA) were not enrolled. However, some patients with a final diagnosis of TIA were included in both groups due to persisting stroke symptoms during the first assessment at HDMC or PSC.

### Statistical analysis

We used the software IBM SPSS Statistics Version 29.0.0.0 for analysis. Clinical characteristics of the two groups were compared by Chi squared test for categorical, and *T*-test for continuous variables. Comparison of NIHSS score between the two groups and the various time components were analyzed by Mann-Whitney *U* test. The frequency of thrombolysis and mRS category at 3 months were analyzed by Chi squared test. Significant differences in clinical characteristics between the two groups were analyzed by multivariate logistic regression to examine their potential impact on thrombolytic rate. *p* Value was set to < 0.05 for statistical significance.

### Ethical considerations

The Rural CT study was approved by Regional Ethic Committee (REC 2017/102) and registered at ClicalTrials.gov (NCT03577847). All patients were asked for informed consent by personal communication or by letter. For patients not able to give consent, the closest relative could approve inclusion into the registry.

## Results

A total of 155 patients were registered in the study, 86 in the Rural CT group, 69 in the Reference group. In the Rural CT group, 47% had remotely controlled CT examination and treatment initiated by paramedics. Three out of 43 (7%) patients in the Rural CT group and 8 out of 41 (20%) patients in the Reference group were lost to 3-months follow up. The clinical characteristics of the two groups are presented in Table 1.

**Table 1.** Clinical characteristics of patients.

Characteristic	Rural CT group	Reference group	*p* Value
	(*n* = 86)	(*n* = 69)	
Age, mean ± SD	3.0 ± 12.4	69.5 ± 12.7	<0.05
Male sex – no. (%)	52 (60.5)	42 (60.9)	0.96
Pre-morbid mRS 0–2 no. (%)	80 (93.0)	64 (92.8)	0.69
Medical history			
Atrial Fibrillation/Flutter – no. (%)	9 (10.5)	7 (10.1)	0.53
Hypertension, medically treated – no. (%)	49 (57.0)	30 (43.5)	0.09
Diabetes Mellitus – no. (%)	6 (7.0)	10 (14.5)	0.13
Current smoking^a^ – no. (%)	15 (17.4)	13 (18.8)	0.96
Hypercholesterolaemia – no. (%)	23 (26.7)	17 (24.6)	0.77
Previous Myocardial Infarction – no. (%)	12 (14.0)	10 (14.5)	0.92
Chronic Heart Failure – no. (%)	5 (5.8)	2 (2.9)	0.39
Previous Aortocoronary Bypass – no. (%)	7 (8.1)	4 (5.8)	0.57
Previous Ischemic Stroke – no. (%)	10 (11.6)	6 (8.7)	0.55
Previous Cerebral Hemorrhage – no. (%)	3 (3.5)	1 (1.4)	0.43
Previous TIA – no. (%)	7 (8.1)	3 (4.3)	0.34
Previous Cerebrovascular Event			
<3 months – no. (%)	3 (3.5)	0	
>3 months – no. (%)	14 (16.3)	9 (13.0)	0.24
Ischemic strokes – no. (%)	38 (44.2)	40 (58.0)	0.09
Intracerebral hemorrhage – no. (%)	5 (5.8)	1 (1.5)	
Stroke mimics – no. (%)	43 (50.0)	28 (40.6)	0.24
Examined by paramedic – no. (%)	40 (46.5)	0	
Transported by HEMS – no. (%)	2 (2.3)	7 (10.1)	
NIHSS score all patients – median (IQR)	2 (0–5)	1 (0–3)	<0.05
NIHSS score ischemic stroke– median (IQR)	3.5 (0–7)	1 (0–2)	<0.05

a Current smoking defined as smoking until 3 months prior to stroke.

Patients in the Rural CT group were 4.8 years older and had a slightly higher median NIHSS score than patients in the Reference group. In both groups, the median NIHSS score was low, indicating many minor strokes and also the inclusion of patients with a discharge diagnosis of stroke mimic.

### Time components

**Figure 3.**
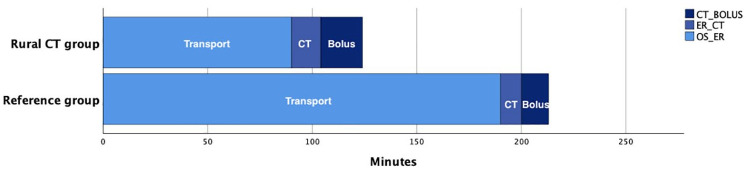
Stacked bar chart illustrating median main time components in patients treated with thrombolysis for the two groups. Time from onset of symptoms to emergency room (Transport), from emergency room to completed CT scanning (CT) and from CT to bolus dose of alteplase (Bolus).

The median time components from onset of symptoms to bolus dose of alteplase are presented in Table 2 and illustrated in Figure 3 and Supplemental Figure 4.

**Table 2.** Median time components in minutes.

Time components	Rural CT group	Reference group	*p* Value
Onset of symptoms – Emergency room^a^ (IQR)	80 (51–121)	205 (131–445)	<0.05
Arrival at emergency room – CT scanning^b^ (IQR)	13 (8–20)	35 (10–109)	<0.05
CT scanning – Bolus of alteplase (IQR)	19 (8–28)	15 (10–38)	0.72
	Rural CT daytime	Rural CT paramedic	*p* Value
Onset of symptoms – Emergency room (IQR)	85 (52–150)	72 (49–104)	0.49
Arrival at emergency room – CT scanning (IQR)	11 (6–20)	16 (13–20)	<0.05
CT scanning – Bolus of alteplase (IQR)	6 (3–25)	19 (17–28)	1.0
	Ref. group daytime	Ref. group on-call^c^	*p* Value
Onset of symptoms – Emergency room (IQR)	193 (116–296)	200 (133–473)	0.35
Arrival at emergency room – CT scanning (IQR)	16 (8–96)	41 (15–108)	0.24
CT scanning – Bolus of alteplase (IQR)	15 (*n* = 1)	13 (10–32)	

a Emergency room: For Rural CT group; arrival at CT lab. Reference group; arrival at the emergency room at PSC.

b CT scanning: Time for completed CT scanning in both groups.

c Ref. group on call: Patients admitted to PSC outside office hours (08 am–04 pm weekdays).

The time from onset of symptoms to emergency call was median (IQR) 20 (8–59) min in the Rural CT group and 60 (13–210) min in the Reference group, *p* = 0.64. The time from onset of symptoms to arrival at the emergency room in the Rural CT group was median (IQR) 80 (51–121) min versus 205 (131–445) min in the Reference group, *p* < 0.05. The time from arrival at the emergency room to completed CT scanning in the two groups was median (IQR) 13 (8–20) min and 35 (10–109) min respectively, *p* < 0.05. The time from CT scanning to bolus dose of alteplase was median (IQR) 19 (8–28) min and 15 (10–59) min, *p* = 0.12. Within the Rural CT group, median (IQR) time from arrival at the emergency room to completed CT scanning was 11 (6–20) min at daytime and 16 (13–20) min in the group handled by paramedics, *p* < 0.05. In the Reference group these median (IQR) times were 16 (8–96) for patients at daytime and 41 (15–108) for patients on-call, *p* = 0.24. In patients treated with thrombolysis, total time from onset of symptoms to bolus of alteplase was median (IQR) 124 min (91–151) in the Rural CT group and 213 min (147–226) in the Reference group, *p* < 0.05, Figure 3.

### Reperfusion therapy, outcomes and complications

Twelve patients (31.6%) with ischemic stroke in the Rural CT group were given intravenous thrombolysis versus eight patients (20.0%) in the Reference group, *p* = 0.24. “Door to needle time” (DTN) in the Rural CT group was median (IQR) 33 (28–39) min versus 21 (20–51) min in the Reference group, *p* = 0.42. We note that age and NIHSS score differed significantly between the two groups in the univariate analysis. These variables and gender were entered into a multivariate logistic regression analysis, but were not significantly related to the thrombolytic rate. Paramedics administered thrombolysis to 6 out of 12 patients in the Rural CT group. Patients performing mRS 0–2 at 3 months follow-up was 54.7% in the Rural CT group and 60.6% in the Reference group, *p* = 0.61. Four patients with stroke mimic received thrombolysis in the Rural CT group versus two in the Reference group, none of them experiencing hemorrhagic complications. In each of the groups, two patients were transferred to the CSC for thrombectomy which was performed in one patient from the Reference group.

One patient in the Rural CT group had symptomatic intracerebral hemorrhage (sICH) following thrombolysis. This patient had a high NIHSS score (24) and an LVO stroke detected on CTA. Following prehospital thrombolysis, the patient was transported to CSC by HEMS for EVT. During CT perfusion imaging a massive ICH was detected, EVT was not performed and the patient died from herniation. This patient received dual antiplatelet therapy due to a myocardial infarction 10 days prior to onset of stroke symptoms.

### Other observed effects

During the study period, seven patients in the rural CT group were diagnosed as stroke mimic after CT scanning, NIHSS and telestroke communication between general physician/paramedic at HDMC and the physician at the PSC, thus avoiding further patient transport. Additionally, two elderly, frail patients with major cerebral hemorrhage could receive palliative treatment at the local nursing home close to their relatives after undergoing rural CT examination.

## Discussion

In the present study, prehospital cerebral CT in a rural medical center was shown to complete a full diagnostic workup 147 min earlier than standard care with transportation to the local hospital. Although the frequency of thrombolysis for ischemic stroke was not significantly higher in the Rural CT group, treated patients received the bolus dose of alteplase significantly earlier. One patient in the Rural CT group suffered from sICH following thrombolysis, while there were no hemorrhagic complications in the Reference group. We do not regard this complication to be specifically related to the rurally performed assessment and treatment.

Outside working hours paramedics assisted in a remote-controlled CT examination and performed diagnostics and treatment including thrombolysis guided by telestroke. When paramedics conducted the CT examination and administration of thrombolysis, more time was spent performing neurological assessment and telestroke guided therapy decision compared to daytime when a general physician was available. This delay was probably related to the cumbersome video-assisted NIHSS scoring procedure. This new method of having trained paramedics performing early prehospital stroke assessment has according to our knowledge not been previously reported nor validated. As long as comprehensive training was mandatory and telestroke guidance was complete, we deemed it safe. Currently, the video-assisted NIHSS scoring by paramedics has been replaced by the introduction of a mobile application, increasing the quality of the assessment.^16,17^ In addition, a future introduction of tenecteplase as thrombolytic agent will further simplify the drug administration.

The substantial time saving from symptom onset to treatment for rurally examined patients was mainly due to a reduction in transport time. The time saved from symptom onset to completed CT enabled an earlier triage and therapy decision. Applying a formula from the metanalysis by Emberson (4, personal communication), the time saving may translate into an increase in odds ratio for good outcome after thrombolysis from 1.32 to 1.58. Furthermore, during office working hours we could perform CTA locally allowing for early identification of patients eligible for thrombectomy. The prehospital time-saving demonstrated in this study may be considered to be of great benefit for the served population. The reduction in time from onset of symptoms to thrombolytic treatment may reduce the number needed to treat (NNT) for good outcome after thrombolysis. On an individual level, the observed time saving may directly impact the degree of disability and quality of future life.

Recent studies have examined the relation between time from onset of symptoms to arrival at hospital and frequency of thrombolysis. A study from Adelaide, Australia, reported higher rate of thrombolysis in patients living closer to stroke centers.^19^ A retrospective cohort study from the U.S. confirmed that rural stroke patients to a lesser extent received intravenous thrombolysis or endovascular therapy and had higher in-hospital mortality than patients from urban areas.^20^

The rural CT model facilitated rapid diagnostics and triage of stroke patients in the served area. Although not reported here, we experienced that other favorable effects of rural CT deployment may be present. Following initial examination and treatment, most patients were transported to RH at low urgency, reducing risks associated with traffic for both the patient and paramedics. Some patients had a rural diagnosis only, avoiding further transport. Finally, some patients with major cerebral hemorrhage could receive local palliative treatment. Apart from acute stroke investigations, the rural CT facility at HDMC is in service in other medical settings completing over 500 outpatient examinations annually.

The significance of rural CT stations like HDMC in acute stroke care have not been extensively studied. However, recent American guidelines describes a similar rural model as Acute Stroke-Ready Hospitals. The role of these is often by telestroke guidance to stabilize the patient, perform a CT scan, provide intravenous thrombolysis and arrange further transportation.^21^

An alternative to rural CT examination is the Mobile Stroke Unit (MSU).^22^ A recent Norwegian study of an MSU model in the semi-rural area of Ostfold demonstrated shortening of time from onset of symptoms to thrombolytic therapy.^23^ Cost may be a limiting factor in the rural application of MSUs.^24^ An analysis from Norway concluded that 260 ischemic stroke patients would need to be treated annually with an MSU to achieve a reasonable cost-effectiveness compared to standard care.^25^ Clearly, a cost-effect analysis of the current Rural CT model at HDMC is needed to advance the program to other rural areas.

### Limitations

We did not perform a randomized trial but established a prospective/retrospective registry with two cooperating hospitals providing data of stroke patients from comparable rural areas. However, within these areas, transport distances and the times from onset of symptoms to emergency call varied widely. In addition, the results should be interpreted keeping the possibility of selection bias in mind. Illustrating this, the patients in the Reference group were younger and had a lower NIHSS score than patients in the Rural CT group. This difference may be attributed to the larger proportion of retrospectively included patients in the Reference group. There is reason to believe that younger patients suffering minor strokes to a greater extent respond to a written request for inclusion. Finally, although examination and treatment at HDMC was the recommended diagnostic acute stroke pathway for patients in the municipalities served by the center, we experienced that many patients were transported directly to RH. The reason for this was mainly logistic decisions from physicians in the most southern municipality in Hallingdal preferring a slightly longer transport time, but thereby gaining direct access to the PSC.

## Conclusions

Our first experience performing CT examination and thrombolytic treatment of stroke in a rurally located district medical center indicate that it may be safe and effective in reducing time from onset of symptoms to revascularization. Paramedics on-site can assist in remote controlled CT examination, perform NIHSS scoring and give intravenous thrombolysis under telestroke guidance from a PSC. Further study is needed to confirm these preliminary findings in a larger patient cohort. Also, a cost effect analysis is needed for wider application of the model. We suggest that a CT laboratory located in a rural district not only provide rapid triage and treatment of stroke patients but generally facilitate diagnosis and treatment at the rural location further optimizing the utilization of healthcare resources. If the necessary infrastructure is provided the model may be applicable to any rural district worldwide effectively increasing access to rapid stroke treatment.

## Supplemental Material

sj-pdf-1-eso-10.1177_23969873241267084 – Supplemental material for Prehospital computed tomography in a rural district for rapid diagnosis and treatment of stroke Supplemental material, sj-pdf-1-eso-10.1177_23969873241267084 for Prehospital computed tomography in a rural district for rapid diagnosis and treatment of stroke by Jørgen Ibsen, Maren Ranhoff Hov, Gunn Eli Tokerud, Julia Fuglum, Marianne Linnerud Krogstad, Marie Stugaard, Hege Ihle-Hansen, Christian Georg Lund and Christian Hall in European Stroke Journal
