# Supplementary material for: Prehospital computed tomography in a rural district for rapid diagnosis and treatment of stroke
Source: Eur Stroke J. 2024 Sep 28;10(1):84–91. doi: 10.1177/23969873241267084 (PMC11556544; doi:10.1177/23969873241267084)
Supplement: sj-pdf-1-eso-10.1177_23969873241267084 – Supplemental material for Prehospital computed tomography in a rural district for rapid diagnosis and treatment of stroke [file sj-pdf-1-eso-10.1177_23969873241267084.pdf]

Supplementary material

A)

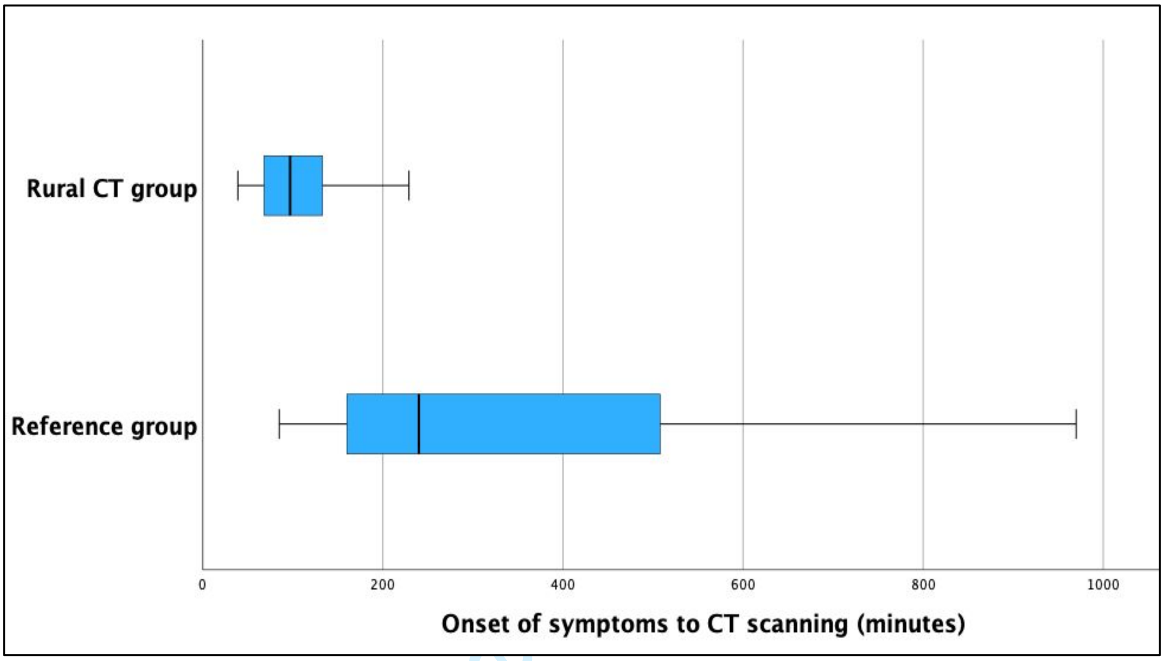

Figure 4. Illustration of time in minutes from onset of symptoms to completed CT scanning for the Rural CT group and the Reference group. The blue boxes represent IQR and min/max values are illustrated by black lines.

B) Video demonstrating the model:

https://www.youtube.com/watch?v=poL9AfsIAhs

eer Review
